# Risk assessment of fungicides on symbiotic phase of arbuscular mycorrhizal fungi

**DOI:** 10.1007/s10646-026-03059-y

**Published:** 2026-03-04

**Authors:** Gilvani Carla Mallmann, Daniela Tomazelli, Leticia Scopel Camargo, Sonia Purin da Cruz, Luís Carlos Iuñes de Oliveira Filho, José Paulo Sousa, Osmar Klauberg-Filho

**Affiliations:** 1 Universidade do Estado de Santa Catarina, UDESC Lages, Lages Santa Catarina, Brazil; 2 Universidade Federal de Santa Catarina, UFSC Curitibanos, Curitibanos Santa Catarina, Brazil; 3 Universidade Federal de Santa Catarina, UFSC Florianópolis, Florianópolis Santa Catarina, Brazil; 4 Centre for Functional Ecology, Associate Laboratory TERRA, Department of Life Sciences, University of Coimbra, Coimbra, Portugal

**Keywords:** *In vivo* tests, ecotoxicological assay, intermediate tier, chlorothalonil

## Abstract

The widespread use of plant protection products (PPPs) may lead to soil contamination, potentially compromising the symbiotic integrity of arbuscular mycorrhizal fungi (AMF) in agricultural systems. However, the effects of PPPs on AMF are underexplored due to the absence of standardized methodology for ecotoxicological assessments. The objective of this study was to introduce an in vivo method for assessing the effects of PPP pollutants on the AMF symbiotic phase and to evaluate the suitability of this method as an intermediate-tier protocol in risk assessment frameworks. Four tests were conducted using combinations of: (1) *Gigaspora albida* + *Glycine max*; (2) *G. albida* + *Urochloa brizantha*; (3) *Rhizophagus clarus* + *G. max*; (4) *R. clarus* + *U. brizantha*). All assays were performed in tropical artificial soil (TAS) under a gradient of chlorothalonil concentrations (0, 12, 18, 24, 36, 48, and 72 mg a.i. kg⁻^1^). The evaluated endpoints included total root colonization, percentage of arbuscules colonization, total extraradical mycelial length (ERM), and spore number. All endpoints were sensitive to the presence of PPPs in TAS, with mycorrhizal colonization and ERM being the most sensitive, meeting the validity criteria (CV < 30%). The Inhibitory concentration (IC_50_) values for all endpoints were higher than the predicted environmental concentrations (PECs). Therefore, this method can be considered suitable as an intermediate-tier protocol, as it exhibits key characteristics of a standardized approach and can be applied to ecotoxicological studies involving other potentially contaminating PPPs, as well as additional classes of environmental contaminants.

**Supplementary Information:**

The online version contains supplementary material available at 10.1007/s10646-026-03059-y.

## Introduction

The use of plant protection products (PPP) has historically been essential to increase agricultural productivity and meet global food demands (Li 2021; Faburé et al. 2025). However, the intensive and widespread application of these compounds may lead to soil and water contamination through the transformation, accumulation, and transport, especially of persistent molecules (Tudi et al. 2021), thereby compromising the life cycle of microorganisms that are essential for plant nutrition and the provision of ecosystem services (Holt et al. 2016; Bertrand et al. 2025). Arbuscular mycorrhizal fungi (AMF) are obligate symbionts that depend on host plants to complete their life cycle (Van der Heijden et al. 2015). The importance of this mutualistic symbiosis for ecosystems is well established, particularly with respect to the nutritional benefits conferred to host plants (Vos et al. 2013; van der Heijden et al. 2015; Rahman et al. 2018). Furthermore, a wide range of additional ecosystem services arises from the mycorrhizal network connecting plant roots within a community, including soil structure stabilization, nutrient cycling, and plant community dynamics (Selosse and Duplessis 2006; Purin and Rillig 2007; Cavagnaro et al. 2015; Rillig et al. 2015; Jansa and Treseder 2016; Antunes and Koyama 2017; Demenois et al. 2018; McGowan et al. 2019).

Despite the ecological relevance of AMF across diverse ecosystems, there is still a lack of comprehensive understanding of the effects of potential pollutants, such as PPPs, on spore germination, extraradical hyphal growth, and intraradical colonization (Twanabasu et al. 2013), particularly concerning impacts on arbuscule formation, the structures responsible for nutrient exchange between plants and fungi. This knowledge gap is particularly concerning, given the widespread use of PPPs in agriculture and their potential to negatively affect AMF performance and the benefits they provide to crop production. In addition, AMF are commercially available and widely applied as agricultural inoculants in Europe, United States and Australia (Koziol et al. 2024, 2025; Salomon et al. 2022) and their effectiveness may be compromised by exposure to chemicals that inhibit spore germination or symbiosis establishment.

These concerns have received increasing attention from regulatory agencies such as the European Food Safety Agency (EFSA), which highlighted the need to develop methodologies capable of assessing the effects of chemical substances across different stages of the AMF life cycle (EFSA PPR et al. 2017). Typically, studies addressing the effects of PPPs effects on the symbiotic phase are conducted using pot experiments with host plants (in vivo system), due to the obligate biotrophic nature of AMF (Hernandez-Dorrego and Pares 2010; Ipsilantis et al. 2012; Karpouzas et al. 2014; Hage-Ahmed et al. 2019). Studies commonly rely on root colonization rate as the primary endpoint, which often results in inconclusive or inconsistent responses. In addition, such studies frequently lack standardization of biotic and abiotic conditions, which are essential to ensure reproducibility and comparability between toxicity assessments. Parameters such as experimental unit size, substrate type, assay duration, and sampling efforts must be clearly defined a priori to enable the development of a standardized method (EFSA PPR et al., 2017). Moreover, additional endpoints beyond total root colonization should be considered, including the percentage of arbuscules, length of extraradical mycelium, and spore production (Hernandez-Dorrego and Pares 2010; Ipsilantis et al. 2012; Karpouzas et al. 2014; Hage‐Ahmed et al. 2019), as these parameters are associated with distinct functional roles of AMF in plant-fungus interactions. Evaluating these endpoints enables a more comprehensive understanding of how PPP exposure may impair AMF functions, ultimately imposing constraints on host plant performance and crop productivity.

Recently, Mallmann et al. (2018) proposed an ecotoxicological protocol to assess the acute effects of PPPs on AMF spore germination during the pre-symbiotic phase. Nevertheless, the effects of PPPs on mycorrhizal symbiosis and its functional attributes remain insufficiently explored, largely due to the lack of standardized experimental procedures, which are addressed and evaluated in the present study. The proposed method enables the assessment of the effects of PPPs on symbiotic-phase-related endpoints of two phylogenetically distinct AMF species (*Gigaspora albida* and *Rhizophagus clarus*) in association with a leguminous crop (*Glycine max)* and a grass species (*Urochloa brizantha*) exposed to the fungicide chlorothalonil (Bravonil^®^ 500, 500 g a.i. L⁻^1^). The hypotheses tested were: (i) the proposed experimental model enables the evaluation of induced effects of PPPs on symbiotic-phase characteristics of AMF; (ii) total root colonization, along with additional parameters such as arbuscule percentage, extraradical mycelium length (ERM) and spore number, are sensitive endpoints suitable for standardized ecotoxicological tests; (iii) both AMF species (*G. albida* and *R. clarus*) are appropriate for use in symbiotic-phase risk assessment protocols, regardless of host plant identity.

## Materials and methods

### Model PPPs and test soil

A commercial formulation of the fungicide chlorothalonil (Bravonil^®^ 500, 500 g a.i. L⁻^1^) was used as the test substance (Table S1). Chlorothalonil is a non-systemic fungicide exhibiting a multi-site mode of action, primarily targeting thiol-containing molecules and impairing fungal respiration (Brazil 2017). Chlorothalonil was selected as a model substance because it remains registered and widely used in Brazil, has well-documented effects on soil microorganisms (Baćmaga et al. 2018), and acts as a contact fungicide, allowing the evaluation of AMF sensitivity to a non-systemic PPP (Azi, Habte & Yuen 1991; Pagano et al. 2023), although its use has been banned in the European Union since 2019 due to human health concerns. It is important to mention that the experimental design and the evaluated parameters are independent of the model compound (chlorothalonil) and can be readily implemented for other PPPs, contributing to the establishment of a standardized protocol for assessing pesticide effects on AMF.

Tropical Artificial Soil (TAS) was used as the test substrate and consisted of 75% fine sand, 20% white clay (kaolin), and 5% coconut coir (Niemeyer et al. 2018). The TAS pH (1:5, soil: water) was adjusted to 6.0 ± 0.5 using calcium carbonate (CaCO_3_). The TAS moisture content was adjusted to 50% of the water-holding capacity (WHC) (ISO 2012), as recommended in standardized soil fauna protocols (Alves et al. 2019). To eliminate any pre-existing organisms, the TAS was submitted to three freezing and defrosting cycles, each lasting 24 h.

### Test organisms

Two species of AMF were used: *G. albida* and *R. clarus*, both originally isolated from tropical soils. These species were selected due to their phylogenetic distances and structural characteristics. Both species had previously been employed in assays targeting the pre-symbiotic phase as proposed by Mallmann et al. (2018).

Pure cultures of both AMF isolates were obtained from the International Culture Collection of Glomeromycota (CICG) (< http://www.furb.br/cicg/%3E). The fungi were propagated in 1.5 kg pots using *U. brizantha* as host plant, grown in soil: sand (1:1) substrate (Stutz and Morton 1996). The resulting soil-based inoculum was stored at 4 °C until use. Soybean (*G. max*, cv. Nidera 5909) and brachiaria grass (*U. brizantha*) were selected as host plants, representing dicotyledonous and monocotyledonous species, respectively, under experimental conditions.

### Test assembly and experimental procedures

Four in vivo trials involving different AMF-host plant combinations were conducted, forming the following test batteries: **Test Battery 1** - *G. albida* + soybean; **Test Battery 2** - *R. clarus* + soybean; **Test Battery 3** - *G. albida* + brachiaria grass; and **Test Battery 4** - *R. clarus* + brachiaria grass. In all test batteries, the same chlorothalonil concentrations were evaluated.

Fungicide concentrations (expressed as mg a.i. kg⁻^1^ dry TAS) were calculated based on the active ingredient (a.i.) content of the commercial formulation added to TAS. The tested concentrations include a non-contaminated control (0 mg kg⁻^1^ dry TAS), and six chlorothalonil treatments (12, 18, 24, 36, 48, and 72 mg a.i. kg⁻^1^ dry TAS). Six replications per treatment were established, resulting in 42 experimental units (EUs) per test battery (Fig. S1). Treatments were arranged in a completely randomized design. The concentrations range was estimated based on previous spore germination assays (data not presented), in which the concentration that promoted 50% inhibition of the germination (IC_50_) values of *G. albida* and *R. clarus*, were 12.1 and 36.2 mg a.i. kg⁻^1^ dry TAS, respectively. Thus, the selected concentrations encompassed the IC_50_ values for both species.

After contamination, TAS moisture was adjusted to 50% of the maximum WHC. Plastic tubes (200 mL) were used as containers for the EUs. At the bottom of each tube 30 mL of sterile vermiculite (autoclaved for 45 min in three cycles) was added to prevent substrate loss. Subsequently, 200 g of TAS was gently added without compaction to maintain adequate aeration within the EUs.

AMF spores were extracted from the soil inoculum using the wet sieving technique (Gerdemann and Nicolson 1963), followed by centrifugation in 20% and 60% sucrose gradients (Daniels and Skipper 1982). Spores with intact walls were selected under a stereomicroscope (Carl Zeiss Stemi 2000-C − 50x). Fifty spores were placed in each EUs within 48 h after extraction. Host plant seeds were sown immediately after spore placement. Prior to sowing, seeds were surface-sterilized in 1% sodium hypochlorite solution (NaClO) for five minutes and subsequently rinsed and stored in distilled water until use. Each EU received two soybean seeds and ten brachiaria grass seeds. After sowing, 20 g of TAS was added superficially to prevent desiccation. Finally, two strands of raw cotton string were inserted to a depth of 3 cm in each EU to enable moisture control by capillarity from containers filled with distilled water (Fig. S2).

The trays containing the EUs were maintained in a climate-controlled room at an average temperature of 25 °C and 12 h light/12 h dark photoperiod. Moisture was monitored every three days by weighing five randomly selected tubes. After 14 days, seedlings were thinned to one soybean plant and two brachiaria grass plants per EU.

After 56 days, corresponding to the period required for root colonization and extraradical mycelium production (Hillis et al. 2008), the test batteries were evaluated. Shoots were cut and separated from roots, which were gently washed under running water. Roots were stored in distilled water prior to the assessment of total mycorrhizal colonization and arbuscular colonization. The remaining substrate (vermiculite and TAS) was homogenized and stored in plastic bags for determination of extraradical mycelium length and spore production. All samples were stored at 4 °C until analysis.

### Endpoints assessment

In each test battery, the following endpoints were determined: percentage of total root colonization, percentage of arbuscules, total spore number, and total extra-radicular mycelium length (ERM). To evaluate the percentage of total root colonization, roots were stained as proposed by Koske and Gemma (1989). Total colonization was determined using the gridline intersect method proposed by McGonigle et al. (1990), considering the presence of any AMF structure inside the roots. For arbuscule assessment, only root fragments containing arbuscules were considered. Spore numbers were determined from a 100 g substrate sample per EU using the wet sieving method (Gerdemann and Nicolson 1963). ERM was quantified following Melloni (1996) and Melloni and Cardoso (1999) methods, adapted for TAS (Supplementary Material and Methods).

### Data analyses

All variables were tested for normality (Shapiro-Wilk) and homogeneity of variances (Bartlett Test). Percentage data were transformed using [(arcsin(√x)/(180 × π)], spore counts using **[**(x + 0.5)^1/2^], and ERM data using [log(x)]. A one-way ANOVA was performed (*p* < 0.05), followed by Dunnett test (*p* < 0.05) to compare each treatment with the control, allowing the determination of the non-observed-effect concentration (NOEC) and the lowest-observed-effect concentration (LOEC). The IC_50_, IC_20_, and IC_10_ were estimated for each endpoint using nonlinear regression models, selecting the best fit based on the r^2^ value (Table S2), in accordance with Environment Canada (EC, 2005), using Statistica Software 10.0 (StatSoft 2011). Comparisons between AMF species were performed using the Behrens–Fisher significance test (generalized likelihood ratio test; *p* ≤ 0.05), comparing IC_50_, IC_20_, and IC_10_ values for each species–host plant combination.

The Predicted Environmental Concentration (PEC) was calculated following FOCUS (1997), as recommended in EFSA guidance documents (EFSA PPR et al. 2017), assuming 5 cm soil incorporation depth, soil bulk density of 1.5 g cm⁻^3,^ and zero crop intercepted (see Carniel et al. 2019 for further details). The simplified equation PEC = A/750, where A is the application rate (g a.i. ha⁻^1^) was applied. Considering the maximum single application rate of Bravonil^®^ 500 for soybean crops (3 L ha⁻^1^; 1500 g a.i. ha⁻^1^), the calculated PEC was 2 mg of a.i. kg⁻^1^ dry soil.

## Results

### Test Battery 1 (*G. albida* + *G. max*) and Test Battery 2 (*R. clarus* + *G. max*)

*G. albida* exhibited a mean total root colonization and arbuscular colonization of 88% and 49%, respectively, in soybean roots under control conditions (without PPPs= exposure). The mean extraradical mycelial length (ERM) was 287.2 cm g⁻^1^ of dry TAS, and the number of spores in TAS was 4.7 spores per 100 g (Table S3).

High IC_50_ values were observed for both total colonization and arbuscules formation (both > 72 mg a.i. kg⁻^1^), followed by ERM (44 mg a.i. kg⁻^1^) (Table 1). For all evaluated endpoints, the no-observed-effect concentration (NOEC) was 12 mg a.i. kg⁻^1^ chlorothalonil, lowest-observed-effect concentration (LOEC) was 12 mg a.i. kg⁻^1^. The coefficient of variation (CV) was of 6.2% for total colonization, 24.6% for arbuscular colonization, 5.2% for ERM, and 58.6% for the spore number (Table 2 and Fig. S3).

*R. clarus* promoted mean total root colonization of 70.8% and arbuscular colonization of 22.4%. This species produced an average ERM of 216.6 cm g⁻^1^ dry TAS and mean spore number of 3 per 100 g of TAS (Table S3). For *R. clarus* the lowest inhibitory concentrations (ICs) were observed for the colonization-related endpoints, with IC_50_ values of 17.5 mg a.i. kg⁻^1^ and 20.9 mg a.i. kg⁻^1^ for total colonization and arbuscular colonization, respectively, in contrast to ERM (> 72 mg a.i. kg⁻^1^) (Table 1). The NOEC was below12 mg a.i. kg⁻^1^ chlorothalonil for total colonization and ERM, 12 mg a.i. kg⁻^1^ for arbuscular colonization, and 72 mg a.i. kg⁻^1^ for the spore number. The LOEC was 12 mg a.i. kg⁻^1^ for total colonization and ERM, 18 mg a.i. kg⁻^1^ for arbuscules and > 72 mg for spore number. The CVs were 22.8% for total colonization, 34.8% for arbuscular colonization, 27.5% for ERM and 55.9% for spore number (Table 2 and Fig. S4).

**Table 1:** Inhibitory concentration values (IC_50_, IC_20_, and IC_10_) for toxicity assays of chlorothalonil at Test Battery 1 (*G. albida* + *G. max*; TB1) and Test Battery 2 (*R. clarus* + *G. max*; TB2)

AMF species	% Total Col.	% Arbuscules	ERM	Spore number
	IC_50_ (mg kg⁻^1^)			
*G. albida*	> 72	> 72	44.0 [39.8–48.1]	7.4 [2.1–12.6]
			Linear	Exponential
			*R* = 0.89	*R* = 0.71
*R. clarus*	17.5 [15.0–21.0]	20.9 [13.1–28.8]	55.1 [43.9–66.3]	> 72
	Hormesis	Logistic	Linear	
	*R* = 0.89	*R* = 0.75	*R* = 0.71	
	IC_20_ (mg kg⁻^1^)			
*G. albida*	**41.2** [28.0–54.5]	31.9 [1.6–62.3]	17.6 [15.9–19.2]	3.1 [1.0–5.1]
	Linear	Linear	Linear	Exponential
	*R* = 0.71	*R* = 0.61	*R* = 0.89	*R* = 0.71
*R. clarus*	**14.7** [12.2–17.1]	10.6 [3.4–17.8]	22.0 [17.6–26.5]	> 72
	Linear	Logistic	Linear	
	*R* = 0.82	*R* = 0.75	*R* = 0.71	
	IC_10_ (mg kg⁻^1^)			
*G. albida*	**20.6** [14.0–27.3]	16.0 [0.8–31.2]	8.89 [8.0–9.6]	2.0 [0.4–3.6]
	Linear	Linear	Linear	Exponential
	*R* = 0.71	*R* = 0.61	*R* = 0.89	*R* = 0.71
*R. clarus*	**7.3** [6.1–8.6]	7.1 [0.6–13.6]	11.0 [8.8–13.3]	> 72
	Linear	Logistic	Linear	
	*R* = 0.82	*R* = 0.75	*R* = 0.71	

IC_50, 20, and 10_ = inhibition concentration corresponding to 50, 20, and 10% of endpoint respectively; CI = confidence interval. % Total Col. = total root colonization percentage; % Arbuscules = arbuscular colonization percentage; ERM = total extraradical mycelium; Spores = number of spores produced. In bold, toxicity differences of pairwise analysis between *G. albida* and *R. clarus* for each host plant, assessed by the Behrens–Fisher significance test (*p* ≤ 0.05)

**Table 2:** NOEC, LOEC, and CV for toxicity assays of chlorothalonil at all Test Batteries

Test	% Total Col.	% Arbuscules	ERM	Spore number
	------------------------------- mg kg⁻^1^ -------------------------------			
	**Test Battery 1**: ***G. albida*** **+** ***G. max***			
NOEC	< 12	< 12	< 12	< 12
LOEC	12	12	12	12
CV (control)	6.2%	24.6%	5.2%	58.6%
	**Test Battery 2**: ***R. clarus + G. max***			
NOEC	< 12	12	< 12	72
LOEC	12	18	12	> 72
CV (control)	22.8%	34.8%	27.5%	55.9%
	**Test Battery 3**: ***G. albida*** **+** ***U. brizantha***			
NOEC	12	12	< 12	18
LOEC	18	18	12	24
CV (control)	15.0%	22.6%	17.7%	24.8%
	**Test Battery 4**: ***R. clarus*** **+** ***U. brizantha***			
NOEC	12	18	< 12	< 12
LOEC	18	24	12	12
CV (control)	20.6%	59.3%	24.2%	11.9%

NOEC = non-observed effect concentration; LOEC = lowest observed concentration; CV = coefficient of variation

### Test Battery 3 (*G. albida* + *U. brizantha*) and Test Battery 4 (*R. clarus* + *U. brizantha*)

*G. albida* resulted in mean total root colonization and arbuscular colonization of 59.2% and 14.8%, respectively. The ERM was 173.7 cm g⁻^1^ of dry TAS, and spore number averaged 3.6 *G. albida* spores per 100 g (Table S3). The IC_50_ values were 69.7 mg a.i. kg⁻^1^ for the total colonization and 69.3 mg a.i. kg⁻^1^ for ERM, followed by arbuscular colonization (17.8 mg a.i. kg⁻^1^) and spore number (16.2 mg a.i. kg⁻^1^) (Table 3). The NOEC was 12 mg a.i. for total colonization and arbuscular colonization, below 12 mg a.i. kg⁻^1^ for ERM, and 18 mg a.i. kg⁻^1^ for spore number. The LOEC was 18 mg a.i. kg⁻^1^ for total and arbuscular colonization, 12 mg a.i. for ERM and 24 mg a.i. for spore number. The coefficients of variation (CVs) were 15% for total colonization, 22.6% for arbuscular colonization, 17.7% for ERM, and 24.8% for spore number (Table 2 and Fig. S5).

*R. clarus* promoted mean total colonization of 50% and arbuscular colonization of 8.8%. The mean ERM of 252.4 cm g⁻^1^ dry TAS, and spore number averaged 9.2 spores per 100 g of TAS (Table S3).The highest IC₅₀ value was observed for total colonization (56.6 mg a.i. kg⁻^1^), followed by ERM (> 53.5 mg a.i. kg⁻^1^), arbuscular colonization (23.8 mg a.i. kg⁻^1^), whereas the lowest IC₅₀ was observed for spore number (10.1 mg a.i. kg⁻^1^) (Table 4). The NOEC was 12 mg a.i. kg⁻^1^ for total and arbuscular colonization and below 12 mg a.i. kg⁻^1^ for ERM and spore number. The LOEC was 18 mg a.i. kg⁻^1^ for total colonization, 24 mg a.i. kg⁻^1^ for arbuscular colonization, and 12 mg a.i. kg⁻^1^ for both ERM and spore number. The CVs were 20.6% for total colonization, 59.3% for arbuscular colonization, 24.2% for ERM, and 11.9% for spore number (Table 2 and Fig S6).

**Table 3:** Inhibitory concentration values (IC_50_, IC_20_, and IC_10_) for toxicity assays of chlorothalonil at Test Battery 3 (*G. albida* + *U. brizantha*; TB3) and Test Battery 4 (*R. clarus* + *U. brizantha*; TB4)

AMF species	% Total Col.	% Arbuscules	ERM	Spore number
	IC_50_ (mg kg⁻^1^)			
*G. albida*	69.7 [42.8–97.0]	17.8 [11.4–24.2]	69.3 [40.2–98.3]	**16.2** [12.3–20.1]
	Linear	Logistic	Linear	Logistic
	*R* = 0.58	*R* = 0.74	*R* = 0.45	*R* = 0.81
*R. clarus*	56.5 [35.9–77.2]	23.8 [16.4–31.2]	53.5 [43.0–63.9]	**10.1** [7.0–13.1]
	Linear	Linear	Linear	Exponential
	*R* = 0.71	*R* = 0.69	*R* = 0.71	*R* = 0.88
	IC_20_ (mg kg⁻^1^)			
*G. albida*	28.0 [17.1–38.8]	9.4 [3.2–15.6]	27.7 [16.1–39.3]	**11.8** [7.1–16.5]
	Linear	Logistic	Linear	Logistic
	*R* = 0.58	*R* = 0.74	*R* = 0.45	*R* = 0.81
*R. clarus*	22.6 [14.4–30.9]	9.5 [6.54–12.5]	21.4 [17.2–25.6]	**3.7** [2.5–4.8]
	Linear	Linear	Linear	Exponential
	*R* = 0.71	*R* = 0.69	*R* = 0.71	*R* = 0.88
	IC_10_ (mg kg⁻^1^)			
*G. albida*	14.0 [8.6–19.4]	6.5 [0.8–12.2]	13.9 [8.6–19.7]	**9.9** [4.8–15.0]
	Linear	Logistic	Linear	Logistic
	*R* = 0.58	*R* = 0.74	*R* = 0.45	*R* = 0.81
*R. clarus*	11.3 [7.2–15.4]	4.8 [3.3–6.3]	10.7 [8.6–12.8]	**2.0** [0.81–3.3]
	Linear	Linear	Linear	Exponential
	*R* = 0.71	*R* = 0.69	*R* = 0.71	*R* = 0.88

IC_50, 20,_ and _10_ = inhibition concentration corresponding to 50, 20, and 10% of endpoint respectively; CI = confidence interval. % Total col. = total root colonization percentage; % Arbuscules = arbuscular colonization percentage; ERM = total extra-radicular mycelium; Spores = number of spores produced. In bold, toxicity differences of pairwise analysis between *G. albida* and *R. clarus* for each host plant, assessed by the Behrens–Fisher significance test (*p* ≤ 0.05)

### Calculations of risk possibilities

Considering a very conservative non refined estimated predicted environmental concentration (PEC) of 2 mg chlorothalonil kg^− 1^ soil and the inhibitory concentrations obtained for the evaluated endpoints, Table 4 summarizes the distances between effect concentrations and the fungicide exposure levels. When IC₁₀ and IC₂₀ values were considered, a greater number of scenarios showed proximity to the PEC, with some cases even indicating overlap between effect and exposure (effect concentration/exposure = 1). In contrast, the IC_50_ values, the endpoint more used for suitability in the statistic curve for all endpoints were at least fourfold higher than the PEC.

**Table 4:** Environmental risk assessment of chlorothalonil fungicide on *G. albida* and *R. clarus* symbiosis with *G. max* and *U. brizantha.* Different endpoints were considered, and a PEC of 2 mg a.i. kg⁻^1^ soil

Endpoint	Estimated distance between endpoint and PEC*											
	G. max						U. brizantha					
	G. albida			R. clarus			G. albida			R. clarus		
	IC_50_	IC_20_	IC_10_	IC_50_	IC_20_	IC_10_	IC_50_	IC_20_	IC_10_	IC_50_	IC_20_	IC_10_
% Total col.	36	21	10	9	7	3.6	35	14	7	28	11.3	5.6
%Arbuscules	36	16	8	11	5	3.5	8.9	4.7	3.2	12	4.7	2.4
ERM	22	9	4.5	27.5	11	5.5	35	14	7	27	11	5
Spores	4	1.5	1	36	36	36	8.1	6	4.9	5	1.8	1

*PEC = A/750 = 1500/750 = 2 mg of a.i. kg⁻^1^ dry soil. % Total col. = total root colonization percentage; % Arbuscules = arbuscular colonization percentage; ERM = total extra-radicular mycelium; Spores = number of spores produced

## Discussion

Environmental contamination by chemicals used in agriculture is a global concern that requires careful evaluation, as adverse effects may extend to soil, water, animal, and human populations. The transition toward more sustainable agricultural systems and the preservation of environmental resources depend on ecotoxicological studies capable of identifying potential effects of plant protection products (PPPs) at different biological levels. However, the absence of standardized and reproducible methodologies remains a critical constraint for elucidating pesticide effects on soil fungi, particularly arbuscular mycorrhizal fungi (AMF). Within this context, and considering the objectives of this study, the experimental models proposed here (test batteries) allowed the assessment of chlorothalonil effects on endpoints related to the symbiotic phase establishment of *G. albida* and *R. clarus*.

These AMF species have previously been demonstrated to be suitable for bench-top ecotoxicological assays targeting the pre-symbiotic phase, including spore germination, when exposed to fungicides (mancozeb, chlorothalonil, metalaxyl), insecticides (imidacloprid, thiamethoxam), and herbicides (glyphosate, diuron) (Mallmann et al. 2018; Malfatti et al. 2021, 2023). Their use in the present study extends this applicability to the symbiotic phase, contributing to the development of a more comprehensive and standardized ecotoxicological framework for AMF.

*G. albida* is recognized as a generalist species with low host specificity and widespread occurrence across Brazilian regions (Ceola et al. 2021). Its ecological plasticity allows it to inhabit a diverse array of ecosystems, including temperate grasslands (Bentivenga and Hetrick 1992), tropical and subtropical savannas (Uhlmann et al. 2004), deserts (Dhillion et al. 1995), agroecosystems (Hetrick et al. 1984), and coastal dunes (Stürmer and Bellei, 1994). Similarly, *R. clarus* is considered a cosmopolitan species (Stürmer et al. 2018), which enhances the potential reproducibility and representativeness of the proposed protocol across different soils and geographic regions. These two species belong to phylogenetically divergent groups and exhibit contrasting strategies of biomass allocation between intra-radical colonization and soil exploration (Hart and Reader 2002). Despite these differences, both species were sensitive to chlorothalonil exposure, showing reduced capacity for mycorrhizal colonization and symbiosis establishment.

The observed decline in intraradical colonization and arbuscule abundance indicate a disruption in the structural development of the arbuscular mycorrhizal association. However, the endpoints assessed herein - total root colonization, arbuscular colonization, and extraradical mycelium (ERM) length - quantify the morphological extent of the symbiosis rather than its metabolic functionality. Since trypan blue staining does not distinguish between metabolically active and senescent fungal tissues, these results should be interpreted primarily as indicators of symbiosis establishment. Consequently, implications for nutrient exchange and water uptake are inferred from established structure-function relationship. To more precisely assess functional impairment, future studies should incorporate complementary approaches, such as vital staining techniques (e.g., nitro blue tetrazolium to assess succinate dehydrogenase activity) or biochemical biomarkers of fungal biomass (e.g., neutral lipid fatty acids: NLFA).

The effects of chlorothalonil were pronounced for most endpoints, except for spore number, which exhibited high coefficients of variation (CV), particularly in Test Battery 2. While ERM is widely reported as being structurally associated with plant photosynthesis, nutrient acquisition, and soil carbon sequestration (Gavito et al. 2019; Tomazelli et al. 2022), sporulation is known to be highly variable and influenced by environmental conditions, host plant status, and stress-related responses of the fungus (Castelli et al. 2014). Moreover, sporulation primarily represents a reproductive strategy often decoupled from direct provision of ecosystem services (Oehl et al. 2017; Turrini et al. 2018). The inherent variability of this trait suggests that spore abundance lacks the robustness required to serve as a primary endpoint in standardized ecotoxicological protocols targeting the symbiotic phase.

Crucially, root colonization and ERM do not necessarily serve as direct surrogates for total AMF biomass. Previous studies have demonstrated that fungicide-induced reductions in root colonization do not consistently correlate with decreases in fungal biomass assessed via NLFA biomarkers (Marshall et al. 2011; Olsson and Lekberg 2022). While this underscores the value of biomass data for understanding fungal persistence and resource allocation, biomass-based methodologies entail complex analytical procedures and are not yet integrated into regulatory ecotoxicological guidelines. Consequently, colonization-related endpoints and extraradical mycelium remain the most practical and relevant indicators for standardized, intermediate-tier assessments, as supported by Pagano et al. (2023).

In all fungus–host plant combinations assessed, we successfully determined inhibitory concentrations (IC), as well as NOEC and LOEC values - cornerstone parameters for the ecological risk assessment of PPPs (Sweeney et al. 2022). The inherent ecological plasticity and low host specificity of AMF facilitate their association with a broad spectrum of mycotrophic plants, offering flexibility in host selection for ecotoxicological assays. Nonetheless, the magnitude of chlorothalonil-induced effects varied across specific fungus–host pairings, highlighting species-specific physiological sensitivities and the modulation of symbiosis development by host identity (Hage-Ahmed et al. 2019).

In line with ISO recommendations for plant-based toxicity tests (ISO, 2012), the establishment of a defined list of host plants is essential for protocol standardization. Host species of agronomic relevance, such as *Sorghum bicolor*,* Triticum aestivum*,* Hordeum vulgare*,* Zea mays*, and *Solanum lycopersicum*, are already recommended in ISO 11269-2 and OECD 208 guidelines (OECD 2006; ISO 2012). The present study supports the inclusion of soybean and brachiaria grass as suitable host plants for tropical and subtropical regions.

Regarding experimental validity, soybean assays yielded high precision (CV < 10%) for specific endpoints, whereas brachiaria grass consistently maintained CV values below the 30% threshold across a broader range of parameters, underscoring its suitability as a host for standardized ecotoxicological testing. Furthermore, the ubiquity and accessibility of brachiaria seeds in tropical and subtropical regions (Keller-Grein et al. 1996) reinforce its potential for methodological standardization. Soybean remains a critical test species due to its status as a global agricultural commodities (Holtman et al. 2022) subject to intensive management regimes. Its cultivation entails the extensive application of PPPs that inevitably reach the soil matrix (Washuck et al. 2022), including herbicides (e.g., glyphosate, 2,4-D, atrazine), fungicides (e.g., mancozeb, chlorothalonil), and insecticides (e.g., acephate) (IBAMA, 2024).

In the context of ecological risk assessment, the proximity between predicted environmental concentrations (PEC) and effect concentrations for some endpoints may indicate potential risk under worst-case exposure scenarios. Although regulatory calibration factors for AMF are still lacking, the ratio between effect concentrations and PEC provides a preliminary indication of risk magnitude and supports the relevance of including AMF in soil risk assessment frameworks.

Overall, the effectiveness of the proposed ecotoxicological protocol was supported by the coefficients of variation observed in control treatments, with 75% of the endpoints presenting CV values below 30%.

This consistency indicates low variability under controlled conditions and supports the use of total root colonization and ERM as potential structural indicators for assessing PPP effects on AMF symbiosis establishment. The protocol proposed here balances ecological relevance and experimental feasibility by integrating a symbiotic plant–AMF system under controlled conditions. Future studies may build upon this framework by incorporating metabolic or biomass-based endpoints, testing additional AMF species and host plants, and expanding its applicability to different classes of PPPs.

## Conclusions

Our results demonstrate that the proposed Test Battery and evaluated endpoints constitute a robust standardized method for assessing the ecotoxicity of plant protection products (PPPs) on the AMF symbiotic phase. The methodology validated herein exhibits high versatility, allowing for the evaluation of diverse chemical substances and AMF–host plant combinations. specifically, this approach: (1) ensures the controlled interaction of test organisms under standardized conditions; (2) enables the quantitative assessment of effect magnitude; (3) confirms the high sensitivity of symbiosis-related endpoints, particularly mycorrhizal colonization and ERM, to soil contamination; and (4) facilitates the derivation of toxicity thresholds (ICx) by applying statistical models standard in terrestrial ecotoxicology. Consequently, this protocol fills a critical gap in current risk assessment strategies for AMF symbiotic phase and is suitable for integration into future risk assessment frameworks as an intermediate-tier assay.

## Supplementary Information

Below is the link to the electronic supplementary material.

Supplementary Material 1

Supplementary Material 2
