# Supplementary material for: Risk assessment of fungicides on symbiotic phase of arbuscular mycorrhizal fungi
Source: Ecotoxicology. 2026 Mar 4;35(4):75. doi: 10.1007/s10646-026-03059-y (PMC12960399; doi:10.1007/s10646-026-03059-y)
Supplement: Supplementary file 1 — Supplementary Material 1 [file 10646_2026_3059_MOESM1_ESM.docx]

**Risk assessment of fungicides on symbiotic phase of arbuscular mycorrhizal fungi**

Gilvani Carla Mallmann^1^, Daniela Tomazelli^1^, Leticia Scopel Camargo^1^, Sonia Purin da Cruz^2^, Luís Carlos Iuñes de Oliveira Filho^1,3^, José Paulo Sousa^4^, Osmar Klauberg-Filho^1^*

^1^Universidade do Estado de Santa Catarina, UDESC Lages, SC, Brazil

^2^Universidade Federal de Santa Catarina, UFSC Curitibanos, SC, Brazil

^3^Universidade Federal de Santa Catarina, UFSC Florianópolis, SC, Brazil.

^4^University of Coimbra, Centre for Functional Ecology, Associate Laboratory TERRA, Department of Life Sciences, Coimbra, Portugal

**Supplementary Material and Methods –** 1 to 3

**Supplementary Tables –** 1 to 3

**Supplementary Figures –** 1 to 6

**SUPPORTING INFORMATION - MATERIAL AND METHODS**

1. **Percentage of total and arbuscular AMF colonization**

After being separated from the substrate, the roots were washed to remove all the substrate adhered to them. Samples were kept in distilled water (which must be changed every 7 days if samples remain for prolonged periods without being evaluated). The staining process followed as proposed by Koske and Gemma (1989), with time adjustment for each step. Initially, the roots were immersed in a 10% KOH solution for 50 min in a water bath at 90 °C. After KOH, the root samples were washed thoroughly under running water to remove any residue from the solution. The roots were then placed in a 2% HCl solution and kept in this solution for 60 min. The HCl solution was removed and the samples covered with an acidified glycerol solution (500 ml glycerin, 450 ml distilled water, 50 ml 1% HCL) containing 0.05% trypan blue (0.5 g in 1 L of solution). Between removal of HCl and immersion in trypan blue solution, the samples were not washed. Once immersed in the trypan blue solution, the roots were left in a water bath at 90 °C for 60 minutes, removed from the water bath and left in the same solution overnight. The following day, the root samples were removed from the dye solution, washed superficially to remove excess solution and placed in containers containing distilled water. The stained roots were kept in a refrigerator at 4 °C until the slides were mounted.

For each EUs, ten segments of stained roots with approximately 1 cm were placed on microscope slides. The percentage of total colonization followed that proposed by McGonile et al. (1990). The structures were observed under a microscope at 400x magnification, determining their presence or absence in 50 observed points. The colonized point of the fragment was considered to be the one that showed the presence of at least one fungal structure (hyphae, vesicle, arbuscule or spore). As an endpoint, only roots with the presence of arbuscules were considered. The percentage of colonization was calculated based on the relation between the number of colonized fragments to the total number of fragments observed under the microscope.

Koske RE, Gemma JN (1989) A modified procedure for staining roots to detect VA mycorrhizas. Mycol Res 92:486–505. https://doi.org/10.1016/S0953-7562(89)80195-9

McGonigle TP, Miller MH, Evans DG, Fairchild GL, Swan JA (1990) A new method which gives an objective measure of colonization of roots by vesicular-arbuscular mycorrhizal fungi. New Phytol 115:495–501. https://doi.org/10.1111/j.1469-8137.1990.tb00476.x

1. **Total number of spores**

For each treatment and replicates, a 100 g sample of substrate was used to determine the number of spores. The spores were extracted from the substrate using the wet sieving method (Gerdemann and Nicolson 1963). The sample was placed in a 2 L plastic beaker, to which tap water (approximately 1.7 L) was added and shaken vigorously with a glass rod. After shaking, the suspension underwent a decantation process and, after this period, the supernatant was transferred to two overlapping sieves (300 μm and 53 μm meshes), taking care that the majority of substrate particles were retained in the beaker. The process of stirring (for 30 seconds), decanting (for 1 minute) and transferring to the sieves was repeated three times. The material retained in the 53 μm sieve was transferred to a beaker with a capacity of 50 mL, with the smallest amount of water possible. Afterwards, the contents of the beaker were transferred to 50 mL Falcon tubes, containing a sucrose gradient (20% and 60%; 15 mL of each solution). The Falcon tubes were centrifuged for 2 minutes at 2000 rpm to separate heavier particles of the substrate (which settle) from the spores and fine organic particles (which remain suspended in the sucrose). The supernatant of the material contained in the Falcon tubes was poured back onto the 53 μm sieve and the retained material was washed with tap water to remove excess sucrose. The extracted spores were stored in a new Falcon tube and kept in a refrigerator (4º C) until evaluation. The spores present in each sample were counted under a stereoscopic microscope with 50x of magnification.

Gerdemann JYI, Nicolson TH (1963) Spores of mycorrhizal Endogone species extracted from soil by wet sieving and decanting. Trans Br Mycol Soc 46:235–244. https://doi.org/10.1016/S0007-1536(63)80079-0

1. **Extraradicular mycelium length (ERM)**

For each sample, three sub-samples with 10 g wet substrate each were weighed; two used for the extraction of the ERM and the third used for determination of the water content after drying in an oven (105 °C for 24 h). The mycelium extraction followed these steps: (1) in a 1 L beaker, the sample was manually shaken (30 seconds) with a glass stirring rod, in approximately 500 mL of tap water; (2) after a decanting period (1 minute), the suspension was suspended in overlapping sieves, meshes of 1 and 0.25 mm; (3) below the sieves was placed a beaker with a capacity of 2 L, to collect the filtrate to be used in the following steps; (4) manual stirring/filtering operation was repeated three times and reached filtrate volume close to 1500 mL; (5) all filtered volume was stirred for 30 seconds in blender at the lowest speed; (6) after shaking, the filtrate went through decanting of 2 minutes in the container itself and 500 mL of supernatant was filtered in a sieve of 0.045 mm; (7) the material retained in this sieve was transferred to checkered cellulose nitrate membranes, with a diameter of 4.7 cm and porosity of 0.47 μm, containing 64 squares (8 x 8 squared). The sample was arranged on the membrane and filtered liquid with vacuum pump. For the quantification of ERM the membrane with sample, completely dry was placed under a glass blade of 5 x 5 cm, lubricated with a drop of almond oil to facilitate visualization in the optical microscope. The 64 fields in each membrane were evaluated from the observation of hyphae in a reticulate device coupled to one of the microscope eyepieces. The device called 'Eye piece Whiplle disc', consists of a grid of 10 x 10 squares (each square or reticulum has dimensions of 1 x 1 mm). The number of intersections of hyphae with the horizontal lines of this grid was counted under a microscope at the increase of 625 times (25x of ocular; 25x lens);

The length of the total extraradicular mycelium was expressed in centimeters of hypha per gram of dry soil and the number of intersections previously obtained was transformed into mycelium length by the equation followed information contained in Cardoso-Filho (1994), Melloni (1996) and Nogueira (1997), coming up with the final formula of R = [(0,05922*n)/)10 - θ)]*100, where n remains the number of intersections of the hyphae in the horizontal lines of the grid contained in the device and θ corresponds to the amount of water contained in the substrate (measured in the third heavy subsample at the beginning of the process).

Cardoso-Filho JA (1994) Quantificação do micélio extramatricial de Glomus etunicatum e da sua atividade, em simbiose com o milho. Piracicaba, 121 p. [Master dissertation, Escola superior de Agricultura “Luiz de Queiroz”]. https://doi.org/10.11606/D.11.2018.tde-20181127-155823

Melloni R, Cardoso EJBN (1999) Quantificação de micélio extrarradicular de fungos micorrízicos arbusculares em plantas cítricas e endófitos: I. Método empregado. Rev Bras Ci Solo 23:53–58. http://dx.doi.org/10.1590/S0100-06831999000100007.

Melloni RA (1996) Quantificação do micélio extrarradicular de fungos micorrízicos arbusculares em plantas cítricas. Piracicaba, 83 p. [Master dissertation, Escola superior de Agricultura “Luiz de Queiroz”]. https://doi.org/10.11606/D.11.2019.tde-20190821-123740

**SUPPORTING INFORMATION – TABLES**

**Table S1**. Physical-chemical characteristics of chlorothalonil, based on data from PubChem (https://pubchem.ncbi.nlm.nih.gov/) and IUPAC (https://sitem.herts.ac.uk/aeru/iupac/atoz.htm).

| **Characteristic** | **Chlorothalonil** |
| --- | --- |
| CAS  IUPAC name | 1897-45-6  tetrachloroisophthalonitrile |
| Empirical formula | C_8_Cl_4_N_2_ |
| Molecular mass (g mol^-1^) | 265.91 |
| Relative density (g cm^-1^) | 1.8 |
| Solubility (pH = 7) (mg L^-1^ 20 ºC) | 0.81 |
| Log Kow (at 2 0ºC) | 2.94 |
| Henry's Law constant (25 ºC Pa m^3^ mol^-1^) | 2.50 x 10^-2^ |
| Degradation/Dissipation (days)  Soil (20 °C/aerobic) | DT_50_: 386 |
| Field | DT_50_: 27.6 |

**Table S2.** Nonlinear regression models were used to define IC_50_, IC_20_, and IC_10_ values (concentration relative to 50, 20, and 10% of inhibition, respectively) in variables analyzed.

| **Model** | **Inhibition concentration** | **Regression** |
| --- | --- | --- |
| Exponential | IC_50_ | v2=a*exp(log((a-a*0.5-b*0.5)/a)*(v1/x))+b |
|  | IC_20_ | v2=a*exp(log((a-a*0,2-b*0,8)/a)*(v1/x))+b |
|  | IC_10_ | v2=a*exp(log((a-a*0.1-b*0.9)/a)*(v1/x))+b |
| Hormesis | IC_50_ | v2=(t*(1+h*v1))/(1+((0.5+h*v1)/0.5)*(v1/x)^b) |
|  | IC_20_ | **v2=(t*(1+h*v1))/(1+((0.20+h*v1)/0.80)*(v1/x)^b)** |
|  | IC_10_ | **v2=(t*(1+h*v1))/(1+((0.10+h*v1)/0.90)*(v1/x)^b)** |
| Linear | IC_50_ | v2=((-b*0.5)/x)*v1+b |
|  | IC_20_ | v2=((-b*0.2)/x)*v1+b |
|  | IC_10_ | v2=((-b*0.1)/x)*v1+b |
| Logistic | IC_50_ | v2=t/(1+(v1/x)^b |
|  | IC_20_ | v2=t/(1+(0.20/0.80)*(v1/x)^b) |
|  | IC_10_ | v2=t/(1+(0.10/0.90)*(v1/x)^b) |
| Gompertz | IC_50_ | **v2=g*exp((log(0.5))*(v1/x)^b)** |
|  | IC_20_ | **v2=g*exp((log(0.80))*(v1/x)^b)** |
|  | IC_10_ | **v2=g*exp((log(0.90))*(v1/x)^b)** |

Models in bold do not were useful.

**Table S3.** Range of values for each endpoint in controls of each Test Battery.

|  | **Endpoint** | **Low value** | **Mean value** | **Maximum value** |
| --- | --- | --- | --- | --- |
| **Test Battery 1**  (*G. albida* +  *G. max*) | % Total col. | 80 | 88 | 94 |
|  | % Arbuscular | 32 | 49 | 60 |
|  | Spore number | 2 | 4.7 | 8 |
|  | ERM (cm g^-1^ of dry soil) | 261.4 | 287.2 | 311.1 |
| **Test Battery 2**  (*R. clarus* +  *G. max*) | % Total col. | 54 | 70.8 | 96 |
|  | % Arbuscular | 14 | 22.4 | 30 |
|  | Spore number | 0 | 1.6 | 2 |
|  | ERM (cm g^-1^ of dry soil) | 160.4 | 216.6 | 316.0 |
| **Test Battery 3**  (*G. albida* +  *B. brizantha*) | % Total col. | 48 | 59.2 | 70 |
|  | % Arbuscular | 12 | 14.8 | 20 |
|  | Spore number | 2 | 3.6 | 4 |
|  | ERM (cm g^-1^ of dry soil) | 150.8 | 173.7 | 224.1 |
| **Test Battery 4**  (*R. clarus* +  *B. brizantha*) | % Total col. | 42 | 50 | 64 |
|  | % Arbuscular | 4 | 8.8 | 16 |
|  | Spore number | 8 | 9.2 | 10 |
|  | ERM (cm g^-1^ of dry soil) | 193.9 | 252.4 | 398.0 |

**SUPPORTING INFORMATION – FIGURES**

**Fig. S1** Scheme established for each Test Battery

4

Legend:

1 – 30 mL sterile vermiculite substrate;

2 – 200 g TAS;

3 – Spores of the species of interest, extracted and counted;

4 – Experimental units with spores and seeds of the hosts; ready to receive the 20 g of substrate for coverage;

5 – Full trial unit;

6 – Cotton row inserted in the experimental unit and that allow the adjustment of humidity by capillarity;

7 – Container containing distilled water for moisture adjustment.

5

6

7

**Fig. S2** Assembly scheme of the experimental units for the in vivo experiments and the humidity adjustment system adopted


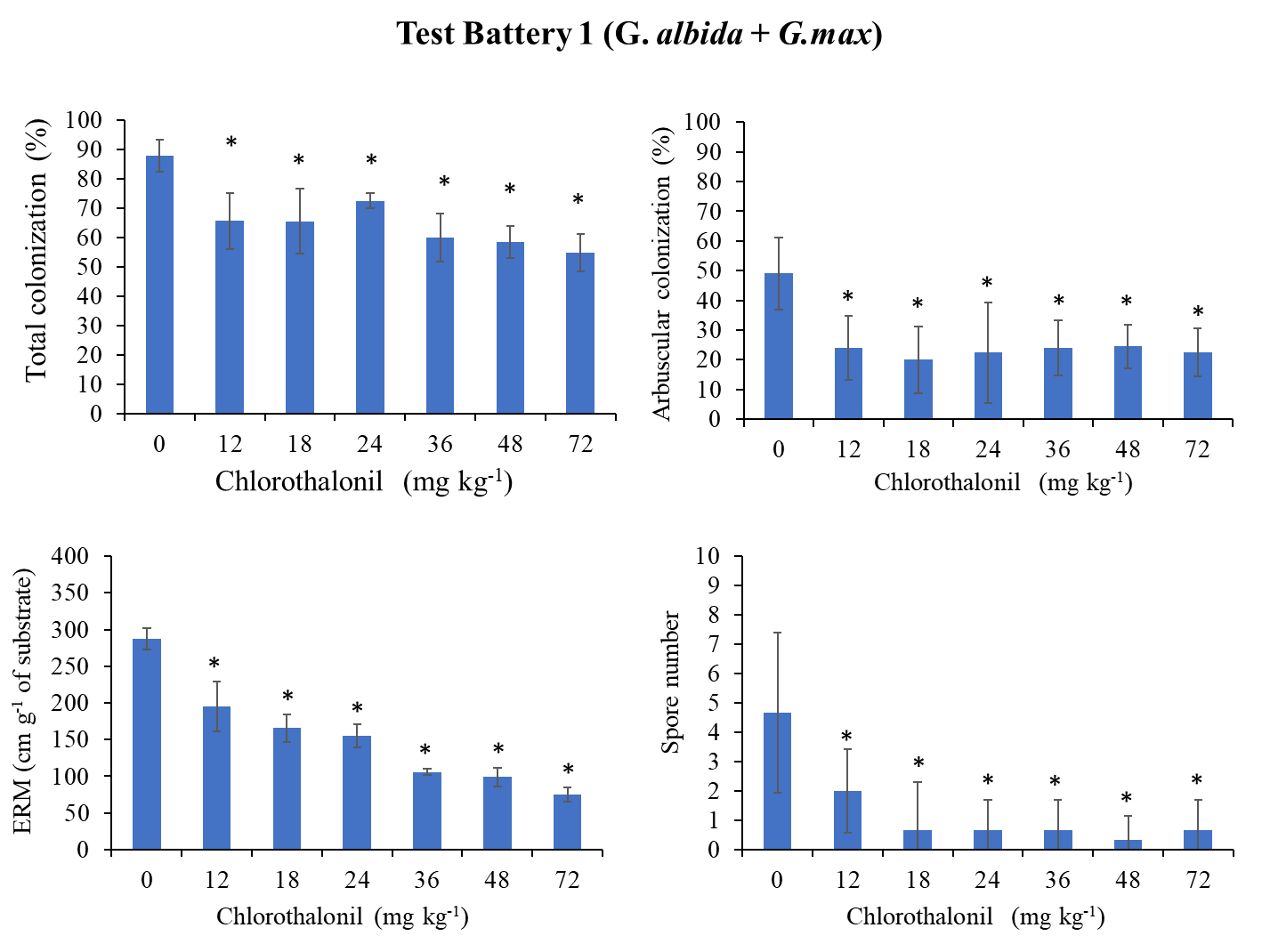
**Fig. S3** Percentage of total and arbuscular root colonization; number of spores per 100 g of substrate and total extra-radicular mycelium length (ERM; cm g^-1^ of substrate) of Test Battery 1 (*G.* *albida* + *G. max*), contaminated with chlorothalonil active ingredient. The data (shown untransformed) is averaged 6 repetitions. Asterisks indicate significant differences in relation to the control by the Dunnett Test (p<0.05). ┬ standard deviation


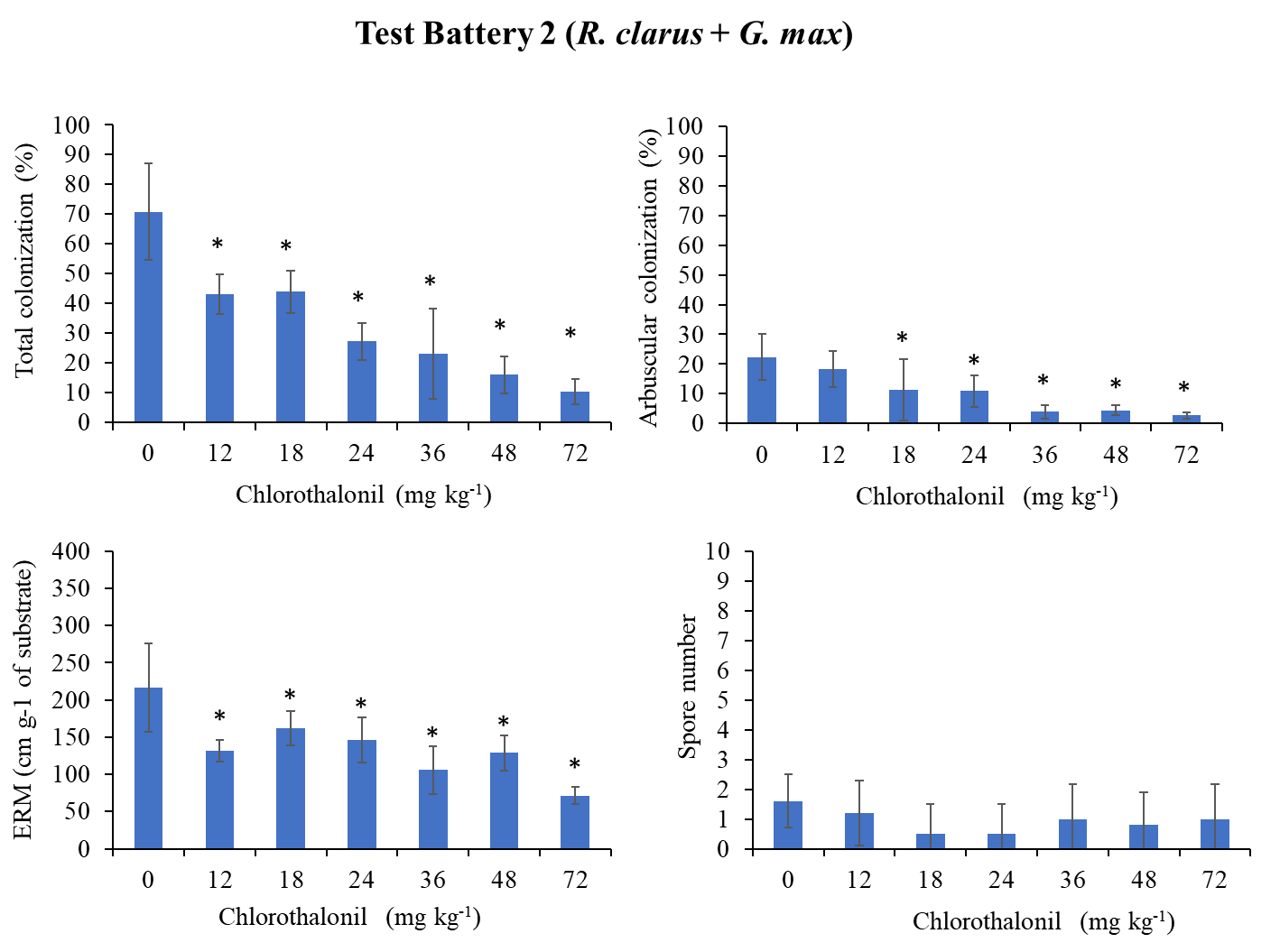


**Fig. S4** Percentage of total and arbuscular root colonization; number of spores per 100 g of substrate and total extra-radicular mycelium length (ERM; cm g^-1^ of substrate) of Test Battery 2 (*R. clarus* + *G. max*), contaminated with chlorothalonil active ingredient. The data (shown untransformed) is averaged 6 repetitions. Asterisks indicate significant differences in relation to the control by the Dunnett Test (p<0.05). ┬ standard deviation

**
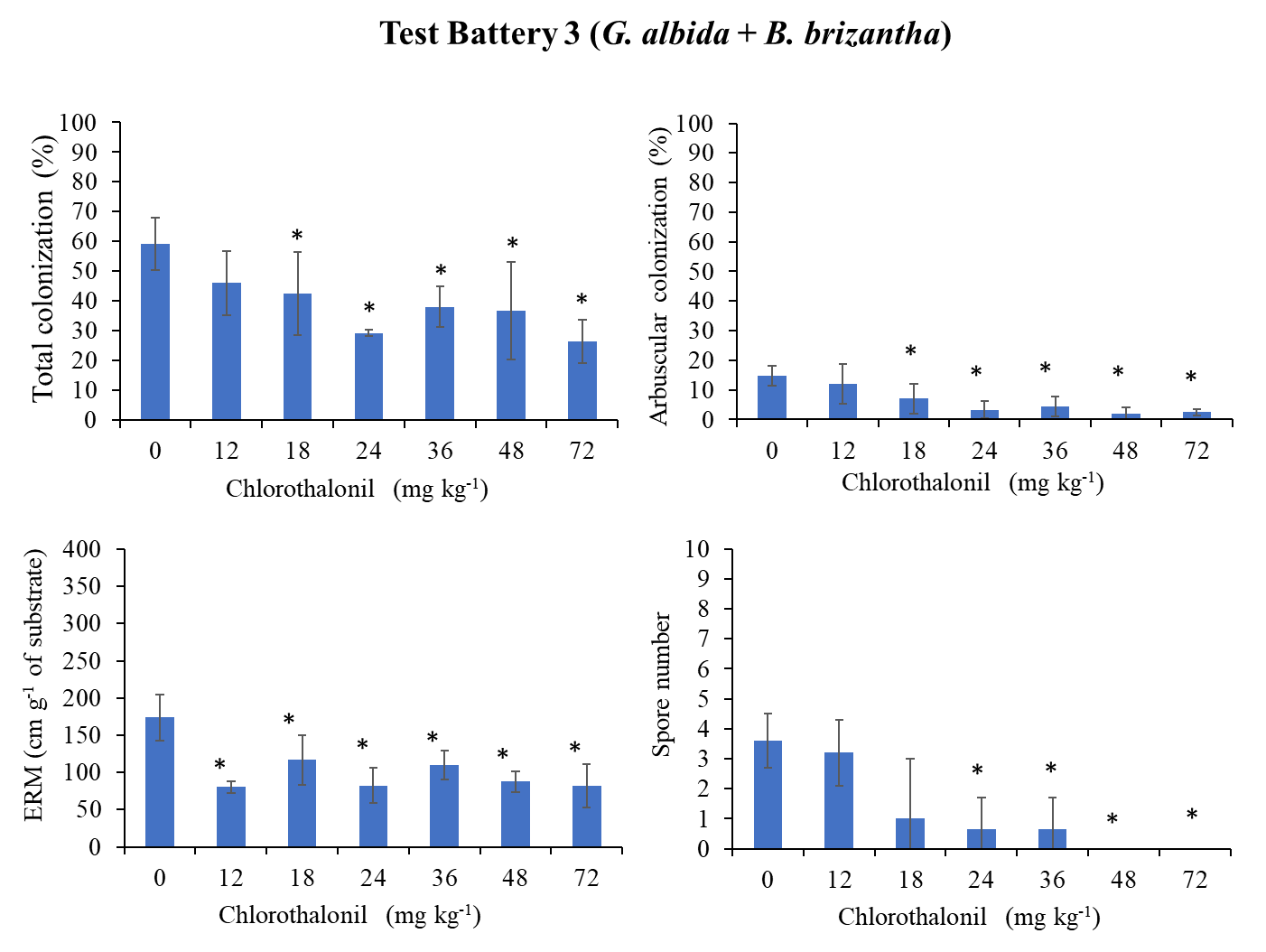
**

**Fig. S5** Percentage of total and arbuscular root colonization; number of spores per 100 g of substrate and total extra-radicular mycelium length (ERM; cm g^-1^ of substrate) of Test Battery 3 (*G. albida* + *B. brizantha*), contaminated with chlorothalonil active ingredient. The data (shown untransformed) is averaged 6 repetitions. Asterisks indicate significant differences in relation to the control by the Dunnett Test (p<0.05). ┬ standard deviation


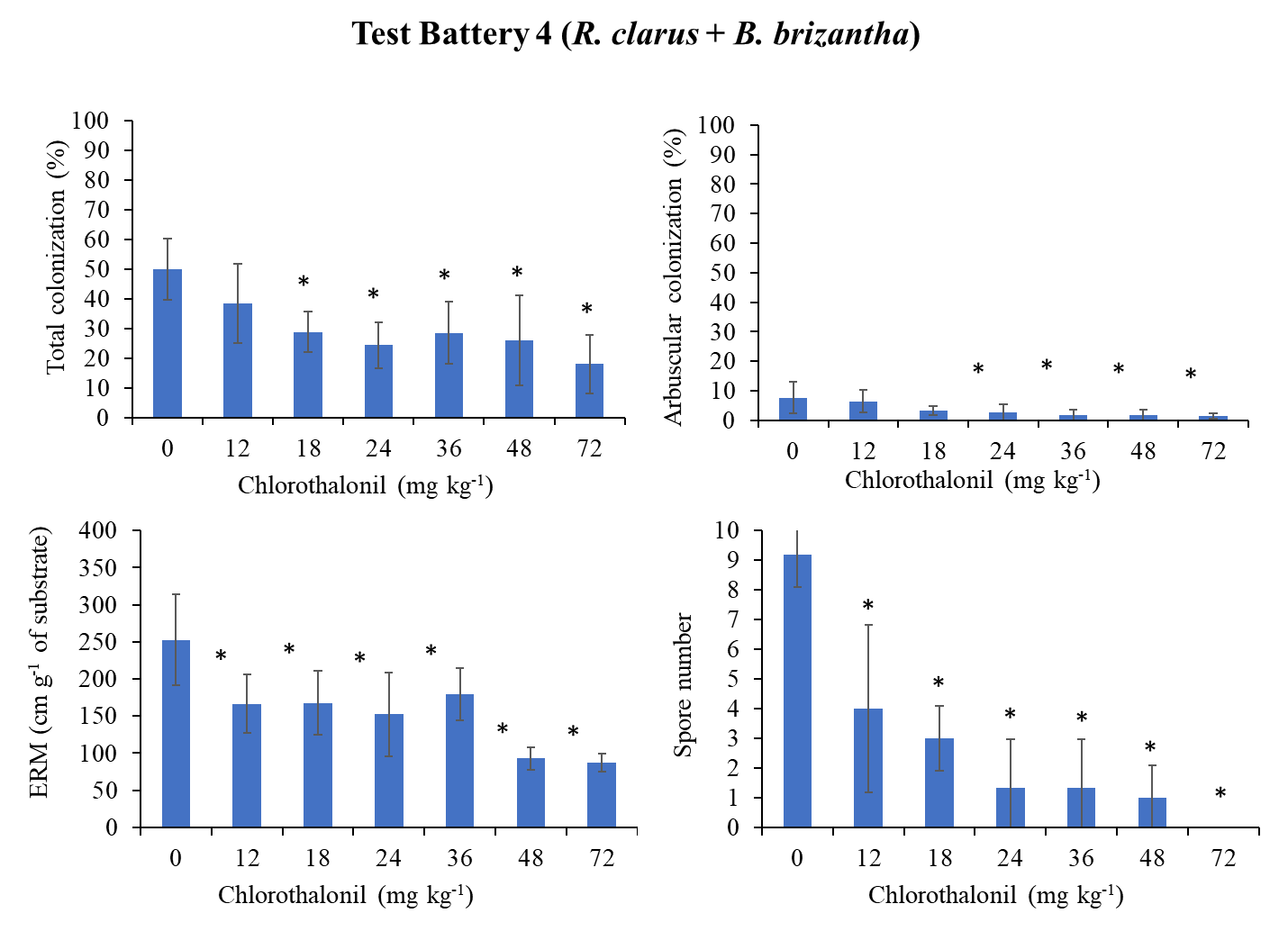


**Fig. S6** Percentage of total and arbuscular root colonization; number of spores per 100 g of substrate and total extra-radicular mycelium length (ERM; cm g^-1^ of substrate) of Test Battery 4 (*R. clarus* + *B. brizantha*), contaminated with chlorothalonil active ingredient. The data (shown untransformed) is averaged 6 repetitions. Asterisks indicate significant differences in relation to the control by the Dunnett Test (p<0.05). ┬ standard deviation
